# Design and Experimental Evaluation of the Proactive Transmission of Replicated Frames Mechanism over Time-Sensitive Networking

**DOI:** 10.3390/s21030756

**Published:** 2021-01-23

**Authors:** Inés Álvarez, Ignasi Furió, Julián Proenza, Manuel Barranco

**Affiliations:** Department of Mathematics and Informatics, University of the Balearic Islands (UIB), 07122 Palma de Mallorca, Spain; ignasi.furio@uib.es (I.F.); julian.proenza@uib.es (J.P.); manuel.barranco@uib.es (M.B.)

**Keywords:** fault tolerance, real-time networks, critical systems, Proactive Transmission of Replicated Frames, Time-Sensitive Networking

## Abstract

In recent years the Time-Sensitive Networking (TSN) Task Group (TG) has been working on proposing a series of standards to provide Ethernet with hard real-time guarantees, online management of the traffic and fault tolerance mechanisms. In this way the TG expects to create the network technology of future novel applications with real-time and reliability requirements. TSN proposes using spatial redundancy to increase the reliability of Ethernet networks, but using spatial redundancy to tolerate temporary faults is not a cost-effective solution. For this reason, we propose to use time redundancy to tolerate temporary faults in the links of TSN-based networks. Specifically, we have proposed the Proactive Transmission of Replicated Frames (PTRF) mechanism, which consists in transmitting several copies of each frame in a preventive manner. In this article we present for the first time a detailed description of the mechanism, with the three different approaches we have designed. We also present the implementation of PTRF in a real TSN prototype. Furthermore, we carry out a qualitative comparison of the different approaches of the mechanism and we experimentally evaluate the approaches of the mechanism in a quantitative manner from three perspectives: the end-to-end delay, the jitter and the bandwidth consumption.

## 1. Introduction

In the last two decades several novel industrial applications have emerged, such as Industry 4.0 systems [1], autonomous vehicles [2] or efficient energy management infrastructures [3]. These applications usually have some common requirements. First, they normally interact with the real world. This imposes tight timing constraints, so that these applications must provide their services in real time, i.e., they must produce their results within a bounded time. Second, they frequently are considered to be critical, as their failure could have catastrophic consequences. Thus, they must be highly dependable in general and highly reliable in particular, i.e., they must provide a correct service throughout a given interval of time with a very high probability.

It is important to note that these novel systems are normally distributed. This makes the communication subsystem fundamental for their correct operation. To that, nodes must be able to exchange information in real time with high reliability. Ethernet is a key technology in this context, given its advantages in terms of high bandwidth, low cost, ubiquity in IP-based networks, know-how and scalability. In fact, Ethernet is probably the technology that has drawn the most attention in recent years not only in data communications but also in the industrial context [1].

Nevertheless, standard Ethernet did not provide real-time (RT) guarantees nor dependability. As a result, a myriad of Ethernet-based industrial communication mechanisms and protocols have been proposed [4]. Each one of these Ethernet-based solutions addresses just one or a subset of the RT and dependability requirements imposed by these novel applications. Certainly, some of these solutions could be combined to build the networks of these new systems, but their high heterogeneity normally imposes strong compatibility or interoperability limitations between communication subsystems that do rely on them.

In order to support RT and dependable communications while overcoming these interoperability limitations, the TSN TG from IEEE has been working to provide a series of technical Ethernet standards [5]. These are commonly referred to as TSN standards and do provide Ethernet with enhanced services in the layer 2 of the network architecture that can be combined to support RT traffic, increased dependability and runtime reconfiguration of the traffic.

For the specific case of reliability, the TSN TG has standardized two mechanisms [6,7,8], which are devoted to deal with faults affecting the channel, i.e., faults affecting links and bridges. The first mechanism consists of a set of spatial redundancy mechanisms that allow transmitting several copies of a frame in parallel, each copy through a different physical path. The second mechanism consists of a set of error-containment techniques included in the bridges to drop untimely or babbling-idiot frames.

Unfortunately, although TSN standards provide these mechanisms to tolerate permanent faults affecting the channel, they do not provide any mechanism specially tailored to tolerate temporary faults. This is an important limitation of the current specification of TSN standards, since temporary faults are more likely than permanent ones. Certainly, spatial redundancy could be used to tolerate temporary faults, but it is not the most suitable solution. As we will show later, if we only use spatial redundancy, we need an extra redundant path for each temporary fault that needs to be tolerated in addition to the permanent ones. This has an important negative impact on the cost of the system, since each additional redundant path requires additional hardware and increases the size, the cost and the energy consumption of the network.

Traditionally, temporary faults in the channel have been addressed by means of time redundancy [4,9], i.e., by means of retransmissions. In the particular case of data networks, retransmissions are normally based on automatic repeat request (ARQ) techniques [10], which rely on acknowledgement (ACK)/negative ACK (NACK) messages and/or timeouts to trigger the retransmission of lost frames. However, ACKs are not well-suited for RT highly reliable industrial networks. First, due to the random nature of temporary faults, ARQ solutions are nondeterministic in terms of end-to-end delay and bandwidth consumption. Second, the end-to-end delay of a frame significantly increases when, and only when, retransmissions are required, which leads to a high jitter in the communications. Third, ARQ solutions introduce additional scenarios involving faults, which are harder to tolerate [11] as temporary faults can also affect ACK/NACKs. Finally, when the network conveys time-triggered (TT) traffic, the schedule must cope with the worst case scenario involving temporary faults. Since ACK/NACKs have to be scheduled in addition to frame retransmissions, ARQ solutions further worsen the utilization efficiency of the bandwidth.

In order to provide TSN networks with adequate mechanisms to tolerate temporary faults affecting the channel, while overcoming the ARQs limitations, we have proposed a time redundancy mechanism called Proactive Transmission of Replicated Frames (PTRF). To the authors’ best knowledge, PTRF is the only time redundancy mechanism proposed for TSN networks so far.

PTRF consists in proactively transmitting several copies of each frame in a preventive manner, to ensure that at least one copy of each frame reaches its destination even in the presence of temporary faults. Note that unlike spatial redundancy, PTRF does not require additional links and bridges. Moreover, PTRF is better suited than ARQ solutions for industrial networks. First, PTRF is deterministic in terms of time and bandwidth consumption, which is specially important for TT RT traffic. Second, proactive replication introduces less jitter, as the time elapsed between frame replicas is as short as the interframe gap. Finally, in the worst-case scenario the end-to-end delay and the required bandwidth are lower than in ARQ solutions, as PTRF does not rely on additional messages (ACK/NACKs) to trigger retransmissions.

More specifically, we have designed three different approaches of PTRF. The first two approaches were introduced in [12] and the third approach was introduced in [13]. Nevertheless, this is the first time that we describe their design and their operation in detail. Moreover, this is the first article that describes their implementation and evaluates their operation through experimentation. Concretely, we evaluate PTRF from three different points of view, (i)end-to-end delay, (ii) traffic jitter and (iii) bandwidth overhead.

The remainder of the document is structured as follows. Section 2 describes the related work of this article while Section 3 provides an overview of the main TSN standards and their most important features for this work. Section 4 describes the basic terminology used when referring to TSN networks and to the PTRF mechanism. Section 5 illustrates with an example how the number of redundant paths increases when we use only spatial redundancy to tolerate temporary and permanent faults, highlighting the need for a time redundancy mechanism. Section 6 thoroughly describes the PTRF mechanism, the fault types and failure modes assumptions, the design rationale, the operation of the three approaches and the structure of the mechanism and the components; while Section 7 presents the implementation of a PTRF device and a fault-injection tool for the evaluation of the mechanism. Section 8 describes the hardware and software that we use to carry out the experimental evaluation of PTRF and Section 9 thoroughly discusses the experiments and the results we have obtained. Finally, Section 10 concludes the paper with a summary of the problem we have addressed and the contributions of this work.

## 2. Related Work

As we have already mentioned, TSN offers a series of fault tolerance mechanisms to tolerate faults at the layer 2 of the network architecture. Concretely, these mechanisms can be used to enforce error containment in the bridges [8] and to create redundant paths to tolerate permanent faults [6,7]. Nevertheless, TSN does not propose any mechanisms in the layer 2 of the network architecture specifically designed to tolerate temporary faults in the links. Thus, temporary faults must be tolerated using spatial redundancy or higher level protocols, such as those based on ARQ [10].

So far we have only talked about spatial redundancy, proactive transmission of replicas and backward error recovery to achieve fault tolerance. Nonetheless, we must note that there are other fault tolerance techniques, such as error correction codes, e.g., erasure codes. Erasure codes consist in codifying frames to transform them in longer frames. The resulting frames convey the original information coded in such a way that the receiver only needs part of the coded frame to recover the original message.

Error correction codes have been extensively used in wireless networks with really high noise, but they are rarely used in wired ones [14]. This is because when the noise is as low as in wired networks, the time required to produce the correction codes and to decode and rebuild frames is considerably higher than the time required for simple retransmissions. Furthermore, in some cases correction codes transmit frames in groups to improve channel efficiency. Nevertheless, the size of the group significantly impacts the time response. This is because frames cannot be transmitted immediately after their creation, but they need to be buffered and transmitted when the whole group is ready. Another common technique to improve the efficiency of correction codes consists in transmitting ACK messages, which, as we have already discussed, introduces a high jitter in the communication. For these reasons, error correction codes are not a suitable solution to tolerate faults in wired networks with hard real-time requirements.

Therefore, we propose to use proactive frame replication to tolerate temporary faults in the links. This technique consists in transmitting several copies of each frame in a preventive manner to maximise the chances that at least one correct copy reaches the destination. Examples of protocols and systems that rely on proactive frame replication to tolerate temporary faults in the links are the Time-Triggered Protocol (TTP) [15], the GOOSE protocol [16], the MARS approach [17] and the Flexible Time-Triggered Replicated Star (FTTRS) network architecture [18].

Even though this is the first article that thoroughly describes the design, implementation and evaluation of the PTRF mechanism, we have published a series of related articles before. In [12] we present a first proposal of the mechanism, introducing two different approaches and comparing them in terms of a preliminary reliability analysis. In [19] we propose a series of mechanisms to integrate the time redundancy provided by PTRF with the spatial redundancy mechanism proposed by the IEEE. In [13] we present a third approach to the PTRF mechanism, we develop a simulation model of the three approaches and we compare the three approaches using exhaustive fault injection.

To the best of the authors’ knowledge this is the first time redundancy mechanism proposed to tolerate temporary faults on TSN-based networks. Moreover, in this article we describe in detail the design of the mechanism for the first time, as well as its implementation on a real platform used by the industry. Finally, we carry out an experimental evaluation to study the mechanism from the time and bandwidth point of view.

## 3. Relevant TSN Standards

As we have mentioned, the TSN TG has developed a series of standards to provide Ethernet with hard and soft real-time capabilities, online traffic management and fault tolerance mechanisms. Each standard proposes a mechanism and these mechanisms can be combined to build the adequate network for each application. Some of the most relevant TSN standards are IEEE Std 802.1AS: Timing and Synchronization for Time-Sensitive Applications [20], IEEE Std 802.1Qav-2009: Forwarding and Queueing Enhancements for Time-Sensitive Streams [21], IEEE Std 802.1Qbv-2015: Enhancements for Scheduled Traffic [22] and IEEE Std 802.1Qcc-2018: Stream Reservation Protocol (SRP) Enhancements and Performance Improvements [23].

The IEEE Std 802.1AS provides global clock synchronisation, required to provide real-time guarantees. The IEEE Std 802.1Qav provides soft real-time guarantees for event-triggered traffic, while the IEEE Std 802.1Qbv standard provides hard real-time guarantees for TT traffic. Finally, the IEEE Std 802.1Qcc allows managing the traffic at runtime. Even though all these standards play a role in the work we have carried out, only the IEEE Std 802.1Qbv standard is relevant to properly describe the experiments we have carried out. Thus, we next describe the main characteristics of this standard. For a detailed description of the other standards, the reader can refer to the respective standards or the official TSN webpage.

**Figure 1:**
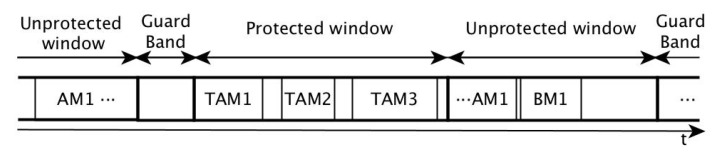
A communication cycle example divided into a protected window, an unprotected window and a guard band.comm-cycle

The IEEE Std 802.1Qbv standard is commonly known as the Time-Aware Shaper (TAS). TAS divides the communication time into slots, called cycles. Each cycle is in turn divided into windows, each window dedicated to a different type of traffic. For instance, the standard shows an example where the communication cycle is divided between a protected and an unprotected window, separated by a guard band to prevent unprotected traffic from interfering with protected one, as shown in Figure 1. In the example, the protected window is devoted to the transmission of traffic with real-time requirements, while the unprotected window is devoted to the transmission of best-effort traffic.

Nevertheless, the communication cycle can be fully customised, i.e., it can be divided in as many windows and traffic types as desired. To achieve this, TAS is based on the *transmission gate* concept. Specifically, TAS proposes the addition of transmission gates to the output queues of the ports. This gate determines whether the frames in the queue can be elected for transmission or not. In this way, TAS can keep the main structure of standard TSN ports untouched, with eight queues, each one corresponding to a different priority, 0 being the lowest and 7 being the highest.

**Figure 2:**
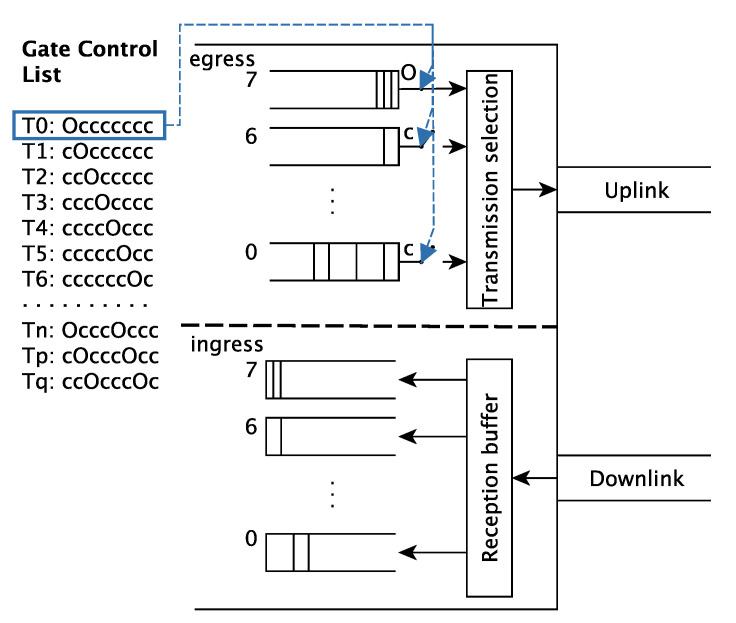
Internal structure of a port with Time-Aware Shaper. Each egress queue of the port has a gate that can be configured as open “O”, to allow frames to be transmitted, or closed “c”, to prevent frames from being transmitted. On the left-hand side of the figure we can see an example of gate control list.

Regarding the operation of TAS, each transmission gate can be in one of two states:(i) open: queued frames can be elected for transmission by the transmission selection algorithm, or (ii) closed: queued frames cannot be elected for transmission. Figure 2 shows the internal structure of a port that implements the TAS. At time instant T0, the gate of the egress queue of priority 7 is open, whereas the other gates are closed.

To provide hard real-time guarantees the gates of all queues in all ports of both nodes and bridges must be properly configured and synchronised. To that, TAS relies on gate control lists. A gate control list is an ordered sequence of gate states which defines the state of each queue during the different windows of the communication cycle. That is, a gate control list dictates when each gate of a port is open and when it is closed, enforcing the desired schedule. On the left-hand side of Figure 2 we see an example of gate control list with the current gate states highlighted in blue.

Furthermore, TAS can be used together with other traffic shapers and transmission policies that rule the transmission of frames within a window. To that, switches implement the shapers and policies, and the adequate rule is selected by the transmission selection algorithm in the port, as shown in Figure 2. For instance, the TSN TG has standardised the Credit Based Shaper (CBS) to provide soft real-time guarantees to the transmission of event-triggered traffic. These mechanisms can be used together to build the appropriate network for each application.

## 4. Terminology

In order to properly describe our mechanism we must first introduce a series of concepts. We start with the description of the different devices that we find in a TSN-based network architecture. The main devices are the end-systems, responsible for executing the application and creating or consuming the information that is exchanged through the network; the bridges, responsible for switching the information from the source to the destination through the established path; and the links, which physically connect end-systems and bridges.

**Figure 3:**
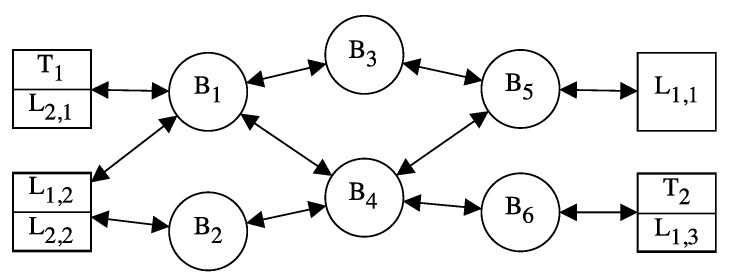
An example of a TSN-based network architecture with four end-systems and six bridges in a mesh topology. End-systems are represented with squares, bridges with circles and links with arrows. The T means that the end-system is a talker, while the L means it is a listener.

Figure 3 shows an example of a TSN-based network architecture. End-systems are represented using squares, bridges are represent with circles and links are represented with arrows. As we can see, there are four end-systems and six bridges, connected in a mesh topology. Furthermore, end-systems can play two different roles: talkers (represented with a T), responsible for producing and transmitting information and listeners (represented with an L), which consume the information created by a certain talker. Each end-system can transmit and consume several flows of information, i.e., an end-system can be a talker and a listener at the same time.

Let us now move to the network-related concepts. A *message* is a piece of information created and transmitted by an application. TSN standards propose the use of *streams* to exchange messages. A stream is a virtual communication channel used to convey traffic with specific characteristics, e.g., traffic with certain period and frame size. Each stream has a single talker, which means that all the traffic transmitted through a specific stream conveys information from the same source, e.g., a temperature sensor or a camera. In fact, the traffic of a stream conveys the same information from the same source captured in different moments, e.g., the value of the temperature in instant $t_{0}$, $t_{1}$, $t_{2}$, etc. We call these the *editions* of a message, or message editions. Whenever an edition of a message must be transmitted, it is embedded together with control information, such as the stream identifier, in a *frame*. A frame is a chain of bits that is injected and transmitted through the physical layer. Therefore, if we want to ensure that an edition of a message is properly delivered to its intended recipients, we actually need to ensure that the frame which contains such message reaches its destination.

We propose to use proactive frame replication, which consists in transmitting several copies of each frame in a preventive manner to maximise the probability of delivering at least one correct copy. Note that each copy of a frame is a frame itself, as it is a chain of bits that is transmitted through the physical layer. In PTRF, the original frame created by the source as well as its copies convey the exact same information, i.e., they are exact replicas. Therefore, we do not differentiate between the original frame and its copies, instead we use the term replica indistinctly throughout the rest of this article. Moreover, whenever we want to refer to a frame that is not replicated by the PTRF mechanism, we refer to it as a nonreplicated frame.

We next describe the experiments carried out to show the drawbacks of only using spatial redundancy to tolerate concurrent permanent and temporary faults and to highlight the benefit of using time redundancy for tolerating temporary faults.

## 5. Spatial Redundancy for Permanent and Temporal Faults

As we have mentioned in Section 1 time redundancy is more suitable than spatial redundancy to tolerate temporary faults in the links. This is because the number of redundant independent paths required to tolerate faults when using only spatial redundancy increases with the number of simultaneous temporary and permanent faults that want to be tolerated. Specifically, in this section we illustrate with an example that the number of paths must be at least one more than the number of simultaneous faults that we want to tolerate. To that, we have carried out four different experiments.

**Figure 4:**
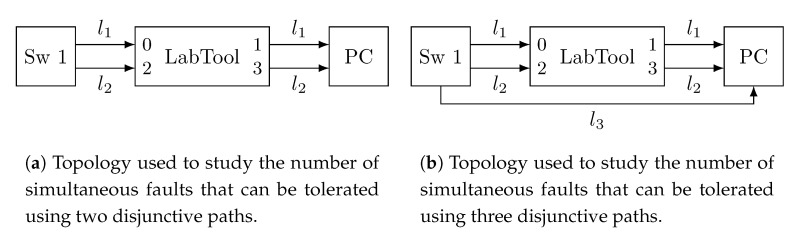
Topologies used to study the number of simultaneous permanent and temporary faults that can be tolerated using spatial redundancy solely.

Figure 4 shows the topologies used for these experiments. Specifically, we use a Multiport TSN (MTSN) switch (indicated in Figure 3 as Sw 1) that acts as an end-system that generates traffic and transmits it through several interfaces. The fault injection device (indicated in Figure 3 as Labtool) is used to inject faults in the links as needed in each experiment. Finally, the PC executes the network analyser tool Wireshark to capture the traffic received through the different links. Figure 4a shows the topology used for experiments I, II and III with two redundant paths; whereas Figure 4b shows the topology used for experiment IV, with three redundant paths. Regarding the traffic configuration, frames transmitted are configured to have priority 5 and frame size of 787 bytes, i.e., 754 bytes of payload plus control information. Furthermore, we transmit 1000 frames through each link in each experiment.

Table 1 shows the number of frames received through each interface of the PC and the number of frames lost in each experiment, i.e., the number of faults that are not tolerated. Experiment I shows the behaviour of the network in the absence of faults. In this experiment the end-system sends 1000 frames through both links and the fault injection device simply forwards the frames with no modifications. We see that the PC receives 1000 frames through each interface, proving the correct operation of the system.

In Experiment II, the end-system sends 1000 frames through both links and the fault injection device injects 1 fault every 100 frames in link 1 and forwards the frames in link 2 with no modifications. This configuration allows us to study the behaviour in the presence of temporary faults in link 1. We can see that the PC receives 990 frames through the first interface and 1000 frames through the second one. Therefore, the temporary faults in link 1 are tolerated using link 2.

In Experiment III, the end-system sends 1000 frames through both links and the fault injection device injects 1 fault every 100 frames in link one, just like in Experiment II. Nevertheless, in this case the fault injection device injects faults in all the frames transmitted through link 2, emulating a permanent fault in the link. In this case, we see that the PC only receives 990 frames through the first interface and none through the second one. Therefore, all the frames affected by a temporary fault in the first link are lost, experimentally proving the rather obvious fact that not all combinations of one permanent fault and a temporary one can be tolerated using only two redundant paths.

Finally, Experiment IV uses the same configuration as Experiment III but with an additional link connecting the end-system to the PC. The third link is fault-free and, therefore, the PC receives 990 frames through the first interface, 0 through the second one and 1000 through the third one, tolerating the simultaneous permanent and temporary faults.

We can see that when considering concurrent permanent and temporary faults, even if the number of temporary faults is low we need to add additional disjunctive paths if we want to guarantee the correct reception of all frames using only spatial redundancy. Nevertheless, the use of spatial redundancy implies a significant increase in the size, cost and energy consumption of the system. Obviously, spatial redundancy is required to tolerate permanent faults, but time redundancy is more adequate to tolerate temporary faults in the links as it does not require a significant increase in the hardware. Moreover, when used together, time redundancy allows taking full advantage of spatial redundancy to tolerate permanent faults, since the latter is not wasted tolerating temporary ones.

We next describe in detail the design rationale of the PTRF mechanism and the operation of the three different approaches of the mechanism.

## 6. The PTRF Mechanism

As we have already mentioned, we have designed the so called PTRF mechanism to tolerate temporary faults in the links of TSN-based networks. Specifically, PTRF is based on proactive frame replication to maximise the chances that at least one copy of each frame reaches its intended destination even in the presence of faults. To do so, PTRF identifies which frames must be replicated and replicates them during their transmission. Upon reception, PTRF identifies and eliminates surplus replicas, ensuring that each frame is only delivered once to the application.

We next describe the fault types and failure modes assumed in this work, we describe the design rationale of the mechanism and we explain the operation and design of PTRF in detail for the first time.

### 6.1. Fault Types and Failure Modes

In order to properly design any fault tolerance mechanism, we must first describe the fault types and failure modes that are expected for the components, in this case, the links. Concretely, we need to specify the types of faults that we want to tolerate with our solution. In this work, our fault model covers temporary nonmalicious hardware faults [24]. That is, faults that last for a finite amount of time, that are caused in an involuntary manner and that only affect the hardware, e.g., electromagnetic interference. This is the type of faults most commonly considered when designing fault-tolerant communication systems.

On the other hand, the failure mode defines how a device may behave in the presence of faults. We assume that links exhibit omission failure semantics, i.e, whenever a fault affects a frame that is being transmitted through a link, the frame becomes erroneous and it is omitted by the system. This is a reasonable assumption since Ethernet frames convey a cyclic redundancy check (CRC) code which allows detecting virtually all errors in the frames upon reception [25]. Erroneous frames are dropped upon reception, manifesting as omissions and thereby preventing the propagation of erroneous information.

### 6.2. PTRF Design Rationale

When designing a system or mechanism, there are virtually infinite ways to carry out such design. In order to produce the design of our mechanism and the resulting three approaches, we have taken into account the main devices and characteristics of TSN-based networks. Specifically, we have considered three aspects: (i) which devices should replicate, (ii) which types of traffic should be replicated and (iii) which is the adequate granularity for deciding the level of replication.

As we have seen, there are two devices in a TSN-based architecture that can carry out the replication of frames, end-systems and bridges. In our designs we have covered all the combinations of devices that replicate: only end-systems replicate, only bridges replicate or both end-systems and bridges replicate.

Regarding the types of traffic, we must note that TSN supports TT and event-triggered traffic with real-time guarantees, as well as best-effort traffic. PTRF is designed to provide time redundancy for critical frames, which are usually related to TT communications. Nevertheless, event-triggered traffic may also convey critical traffic, such as alarms. Thus, the PTRF mechanism can be used to replicate any type of traffic, even best-effort one. It is the responsibility of the network manager or management entity to decide which traffic should be replicated or not and to ensure the timing guarantees of the traffic.

Regarding the granularity of the replication, we have considered two different options. First, we designed the mechanism to allow for a different level of replication for each stream. Nevertheless, when we moved to the implementation phase we encountered an important limitation to this decision. The number of streams in a network is unpredictable and, most likely, high. Therefore, storing and processing information in a per-stream manner required reserving a significant amount of memory and processing resources. To solve this, we have designed PTRF in a way that the level of replication depends on the priority of the stream. We consider this to be a reasonable trade-off, as in TSN streams that share the same priority usually convey traffic with similar characteristics. Thus, we can assume that their reliability requirements are also similar and can share the same level of replication.

Furthermore, we have defined a series of requirements that PTRF must meet. The requirements are the following:

- R1: the mechanism must be fully compatible with standard devices.
- R2: the mechanism must be easily integrated with existing standards.
- R3: the mechanism must not imply significant modifications of standard devices.
- R4: the mechanism must not have a high memory consumption as bridges have a limited amount of memory.
- R5: the mechanism must be flexible enough to be used in virtually any network, even those for dynamic systems.

First, we must note that all the approaches we have designed meet requirements R1 and R2. Regarding R1, even though PTRF requires modifying the devices that carry out the replication of frames and the elimination of surplus replicas, these PTRF devices can coexist with standard devices and PTRF frames can be forwarded by any standard bridge, and vice versa. Regarding R2, we have designed PTRF taking into account the main characteristics of TSN networks. More concretely, we have taken into account the use of streams, priorities and gates to guarantee that our mechanism can be used together with some of the most relevant TSN standards. Unfortunately, we cannot guarantee an easy integration of PTRF with each and every standard proposed by the TSN TG as many of them are unfinished and the number is still growing.

As we have mentioned, we have designed three different approaches. In our first approach, only end-systems replicate frames. This design satisfies requirements R1, R2, R3 and R4. Regarding R3, the modifications are limited to end-systems, which are prone to having custom functionalities, which allows using standard bridges. Regarding R4, since the mechanism is placed in end-systems, bridges do not consume any additional resources. Nevertheless, R5 is not satisfied by this approach, as only nodes can replicate frames and the level of replication is the same for each priority in the whole network.

In our second approach, both end-systems and bridges replicate frames. This approach meets requirements R1, R2, R3 and R4. R3 is satisfied because bridges do not require the modification of existing mechanisms to support PTRF, only the addition of several components which are described in Section 6.4.3. Furthermore, Even though this approach implies the modification of bridges, R4 is satisfied because the amount of additional information that bridges store is not significant, as we use a per-priority replication scheme. Nevertheless, similarly to what happens with the first approach, R5 is not satisfied as all the devices use the same level of replication in the whole network, which varies only depending on the priority.

Finally, we designed a third approach in which both, end-systems and bridges may or may not replicate. That is, all devices can replicate, but they do not necessarily do it. Furthermore, the devices that do replicate can have different levels of replication. This approach meets all the requirements we have defined. Requirements R1 to R4 are met in the same way as in the previous approach, as the components are the same. Nevertheless, in this approach R5 is now met because it supports the full customisation of the devices and their level of replication, as we explain in the next subsection.

Therefore, even though we could explore other design options, the approaches we have designed cover most or all of our requirements. Thus, exploring other designs and their interest is left as future work, e.g., a per-stream replication scheme where only end-systems replicate.

### 6.3. PTRF Operation

As we have already explained, we designed three different approaches to the PTRF mechanism, which differ from each other in the devices that carry the replication out and in the way replicas are handled. We introduced the first two approaches in [12] and the third one in [13]. It is important to note that, even though the approaches have been introduced in said works, we have never described their design and operation in detail before. We next describe each approach and we discuss their advantages and disadvantages.

#### 6.3.1. Approach A: End-to-End Estimation and Replication

In this first approach, only end-systems implement PTRF, i.e., end-systems replicate frames during transmission and eliminate surplus replicas upon reception. Bridges are commercial off-the-shelf (COTS) standard TSN bridges that simply forward all the correct frames that they receive. The number of replicas *k* that end-systems must transmit is decided for the network as a whole using an end-to-end worst-case estimation of frame omission probability. We refer to this approach as approach A in the rest of the article.

**Figure 5:**
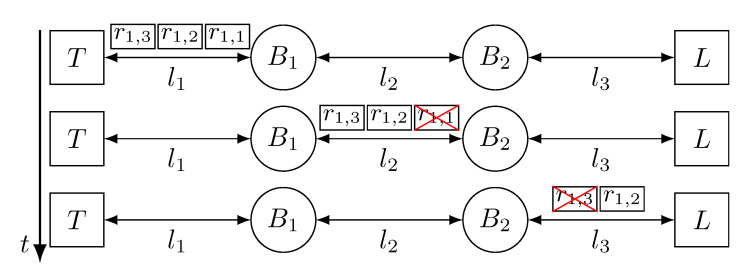
Behaviour of the approach A of the PTRF mechanism in the presence of temporary faults in the links.

Figure 5 depicts the behaviour of a network that uses approach A to tolerate temporary faults in the links. The network has one talker (*T*), one listener (*L*) and two bridges ($B_{1}$ and $B_{2}$), connected by three links ($l_{1}$, $l_{2}$, $l_{3}$) forming a line topology. In this figure, the value of *k* is 3 and, thus *T* transmits three replicas of frame $f_{1}$ ($r_{1,1}$, $r_{1,2}$ and $r_{1,3}$). As we can see, $B_{1}$ receives and forwards the three replicas of $f_{1}$, nevertheless, replica $r_{1,1}$ is affected by a temporary fault in link $l_{2}$, causing its omission. Thus, $B_{2}$ only forwards the correctly received replicas, $r_{1,2}$ and $r_{1,3}$. After that, a temporary fault affects replica $r_{1,3}$ in link $l_{3}$, which is dropped by *L* upon reception. $r_{1,2}$ reaches *L* correctly and it is delivered to the application.

#### 6.3.2. Approach B: End-to-End Estimation, Link-Based Replication

In this approach, both end-systems and bridges implement PTRF, i.e., all network devices replicate frames during transmission and eliminate surplus replicas upon reception. We refer to this approach as approach B in the rest of the article.

As in approach A, the number of replicas $k^{\prime }$ is decided for the network as a whole using an end-to-end worst-case estimation of frame omission probability. Nevertheless, we must note that the value of *k* and $k^{\prime }$ may not be equal for the same network. This is because in approach B all bridges generate a new set of replicas and, thus, the transmission is successful even if $k^{\prime }-1$ replicas are omitted in each link, whereas this is not true for approach A. More details on the relation between *k* and $k^{\prime }$ can be found in [12].

**Figure 6:**
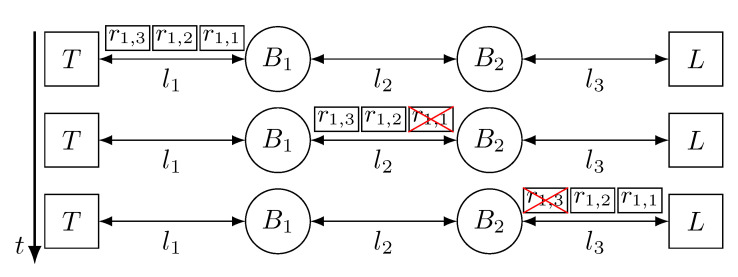
Behaviour of the approach B of the PTRF mechanism in the presence of temporary faults in the links.

Figure 6 depicts the operation of a network that uses approach B to tolerate faults in the links. The network topology and the faults are the same as in Figure 5, but we see that the network behaves differently. Specifically, we can see that, even though $r_{1,1}$ is lost in link $l_{2}$, $B_{2}$ transmits three new replicas through link $l_{3}$. This is because bridges only keep the first correct replica they receive and discard the surplus ones, and, then, they create a new set of replicas during transmission. Again, replica $r_{1,3}$ is affected by a fault in link $l_{3}$, but *L* receives two replicas now, instead of one. Thus, *L* receives replica $r_{1,1}$ first, and delivers it to the application and discards replica $r_{1,2}$.

#### 6.3.3. Approach C: Link-Based Estimation and Replication

In this approach, both end-systems and bridges implement PTRF, i.e., all the devices in the network replicate frames during transmission and eliminate surplus replicas upon reception, as in approach B. Nonetheless, the number of replicas that must be transmitted through each link *m* can vary depending on the loss probability of said link. Therefore, each device may transmit a different number of replicas $k_{m}^{\prime \prime }$ through each link. We refer to this approach as approach C in the rest of the article.

**Figure 7:**
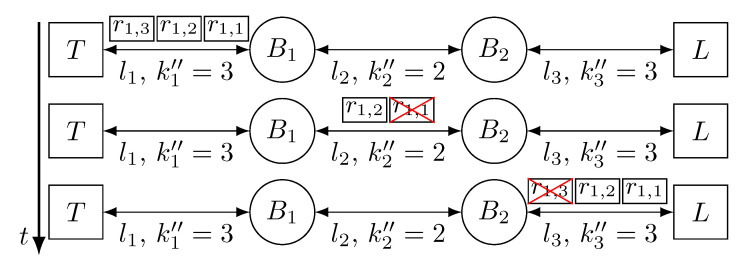
Behaviour of the approach C of the PTRF mechanism in the presence of temporary faults in the links.

Figure 7 depicts the behaviour of a network that uses approach C to tolerate temporary faults in the links. The scenario shown in Figure 7 is the same as in Figure 5 and Figure 6, but we can see that the number of replicas transmitted by the devices varies. *T* transmits 3 replicas through link $l_{1}$, but we see that, even though all replicas are correctly received by $B_{1}$, it only transmits two replicas through link $l_{2}$, as it is configured to do so. Again, replica $r_{1,1}$ is affected by a fault in link $l_{2}$ and, thus, dropped by $B_{2}$. Nevertheless, $B_{2}$ transmits 3 replicas again and, even though replica $r_{1,3}$ is affected by a fault, *L* receives replicas $r_{1,1}$ and $r_{1,2}$, it delivers $r_{1,1}$ to the application and discards $r_{1,2}$.

#### 6.3.4. Qualitative Comparison of the Approaches

Each approach presents a series of advantages and disadvantages. On the one hand, approach A allows using COTS TSN bridges, which can significantly reduce the cost of the network. On the other hand, the number of fault scenarios that can be tolerated before losing information is low compared to the other two approaches. We call a fault scenario to any of the possible combinations of faults that cause the omission of a subset of replicas. Let us assume we have a talker that transmits frame $f_{1}$ with k=2, resulting in the transmission of replicas $r_{1,1}$ and $r_{1,2}$. In this case, the number of fault scenarios that can be tolerated by approach A is two for the whole path, the omission of $r_{1,1}$ or the omission of $r_{1,2}$, as any other scenario would cause the omission of both $r_{1,1}$ and $r_{1,2}$. Nevertheless, when using approach B the number of scenarios that can be tolerated increases with the number of links. This is so approach B can tolerate the loss of any of the replicas in each link, as the following device creates a complete set of replicas again. More details on how to calculate the number of scenarios that can be tolerated by each approach can be found in [13].

**Figure 8:**
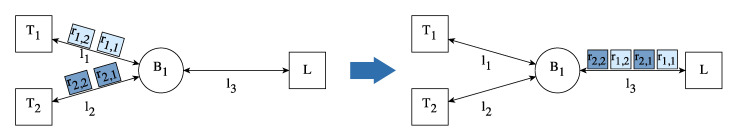
Example of interleaving replicas when using the approach A of the PTRF mechanism to tolerate temporary faults in the links.

Furthermore, in approach A the schedule must be adapted to take into account that there might be interfering frames between the transmission of replicas by the bridges. Figure 8 depicts a scenario in which there is interleaving of replicas when using approach A. In this example we see a network with two Talkers ($T_{1}$ and $T_{2}$), one bridge ($B_{1}$) and a Listener (*L*), connected through 3 links ($l_{1}$, $l_{2}$ and $l_{3}$). Each talker transmits a frame, $f_{1}$ and $f_{2}$ respectively, which are replicated twice, resulting in replicas $r_{1,1}$ and $r_{1,2}$ for frame $f_{1}$ and replicas $r_{2,1}$ and $r_{2,2}$ for frame $f_{2}$. Let us assume that $f_{1}$ and $f_{2}$ have the same priority and, thus, are queued in the same transmission buffer of bridge $B_{1}$. Let us also assume that replica $r_{1,1}$ reaches bridge $B_{1}$ and is forwarded to the egress port. Then, replica $r_{2,1}$ reaches bridge $B_{1}$ next and is forwarded to the egress port before replica $r_{1,2}$. As we can see, this situation can easily happen when frames with the same priority are transmitted through different network paths. Therefore, if this is not properly reflected in the scheduling, the last replicas of a frame may violate the deadlines calculated for the first one.

Regarding approach B, it allows tolerating a significantly higher number of fault scenarios than approach A when $k^{\prime }$ = *k*, as we proved in [13]. Moreover, we must note that the design of this approach prevents interleaving of replicas, as replicas are created in the egress port and they are immediately queued in the transmission buffer. Thus, all the replicas of a frame are created and queued before processing the following frame. This enables the use of existing schedulers which now only need to take into account the time required to create and transmit $k^{\prime }$ replicas instead of a single frame.

Nevertheless, in approach B, all the devices of the network must be modified to support PTRF, which can lead to an increase in the price of the network. Furthermore, approach B can lead to an inefficient use of the network bandwidth, as the number of replicas to be transmitted in each link is calculated using a worst-case estimation for the whole network. Therefore, even if the omission probability is low in certain parts of the network the level of replication must be high enough to tolerate the faults in the areas with harsher conditions.

Approach C is a compromise between approaches A and B. Like in approach B, bridges must be modified to support PTRF and it can tolerate a significant amount of fault scenarios [13]. However, contrary to approach B, approach C allows adapting the number of replicas transmitted according to the vulnerability of each link, thus reducing the bandwidth consumed to tolerate faults while guaranteeing an adequate level of fault tolerance. Furthermore, approach C prevents interleaving of frames between replicas as the replication mechanism is the same as in approach B.

Finally, this approach is specially suitable to support dynamic fault tolerance and to integrate temporal and spatial redundancy [19] in the same network. On the one hand, flexibility is key for adaptive systems and being able to adapt the level of replication depending on the vulnerability of the link provides a high level of flexibility for fault tolerance. On the other hand, in order to combine spatial and time redundancy in an efficient manner, it is important to adjust the level of time redundancy to the availability of spatial redundancy in the network. That is, whenever spatial redundancy is available, time redundancy can be deactivated to save resources.

### 6.4. PTRF Design

In order to support the PTRF mechanism, devices and frames must undergo a series of modifications. We have classified these modifications into three groups, namely (i) functions, (ii) frames and (iii) components. We next describe these modifications in detail.

#### 6.4.1. Functions

**Figure 9:**
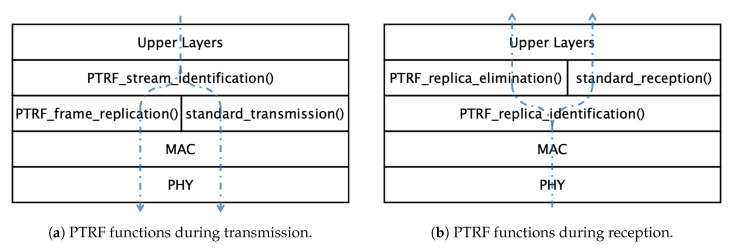
Functions of the PTRF mechanism in reception and transmission.

We have divided the operation of PTRF into functions, which are shown in Figure 9. Specifically, Figure 9a shows the functions needed for the transmission of replicas and Figure 9b shows the functions needed to support the reception of replicas.

We can divide PTRF in two functions during transmission.

- PTRF_stream_identification: this function is responsible for differentiating frames that must be replicated from those that must not. This is done by checking the priority of the frame, as it will be detailed in Section 6.4.3. If the frame must be replicated, the function PTRF_frame_replication is executed, otherwise, the standard transmission process is executed.
- PTRF_frame_replication: this function carries out the replication of frames. First, this function embeds in the frame some information which is important for the correct operation of PTRF, which we detail in Section 6.4.2. Then, the frame is replicated the required amount of times, depending on its priority, and each replica is queued for transmission.

We can divide PTRF into two functions during reception.

- PTRF_replica_identification: whenever a frame is received through a port that implements PTRF, this function detects whether the frame is a replica or not. This is done by looking for the PTRF Ethertype, which is introduced in the frame by function PTRF_frame_replication, as indicated in Section 6.4.2. If the frame conveys such Ethertype, the PTRF_replica_elimination function in executed. Otherwise, the standard reception process is chosen.
- PTRF_replica_elimination: this function is responsible for eliminating surplus replicas. To do so, the function checks whether this is the first replica of a given frame that has been received or not. To this aim, each port counts with a database that stores key information to identify the last received replicas, as we further explain in Section 6.4.3. If this is in fact the first received replica of a given frame, the database is updated and the replica is delivered to the application. Otherwise, the replica is discarded.

#### 6.4.2. Frames

**Figure 10:**
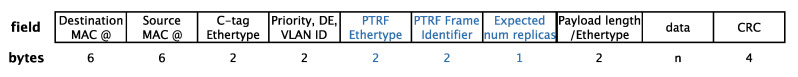
Format of an IEEE 802.1Q Ethernet Data frame that has been replicated using PTRF. As we can see highlighted in blue, the frame conveys new fields, namely the PTRF Ethertype, PTRF frame identifier and the expected number of replicas.

As we have already mentioned, the PTRF mechanism requires the addition of information to frame replicas. Specifically, we add three new fields in each replica. This additional information is conveyed in every replica, but it is not required for the transmission of nonreplicated frames. Moreover, the structure of PTRF replicas is compatible with any TSN or standard Ethernet bridge, as we explain later on. Figure 10 shows the structure of replicas and we describe it next.

- PTRF Ethertype: This field allows differentiating replicas created by the PTRF mechanism from those frames that are not replicated. This field occupies 2 bytes and its value is 0x8815. It is important to note that if the original frame conveys an Ethertype to identify a higher layer protocol, said Ethertype is not deleted, only shifted to the Payload length/Ethertype field so the replica can be properly processed upon reception.
- PTRF frame identifier: This field allows distinguishing replicas that do not convey the same information. As we explain in Section 4, the frames transmitted through a single stream convey information from the same source produced in different moments, $t_{0}$, $t_{1}$, $t_{2}$, etc. As we explained in Section 4, we call these message editions. To identify replicas, it is important to distinguish whether they belong to the same message edition or not. Nevertheless, the control information of TSN frames is exactly the same for all the frames transmitted through a stream, i.e., frames that convey different message editions carry the exact same control information, only the payload is different. Therefore, we add this field to distinguish the replicas of different message editions, i.e., the replicas transmitted in $t_{0}$ from the ones transmitted in $t_{1}$, the ones transmitted in $t_{1}$ from those transmitted in $t_{2}$, etc. This field is 2 bytes long, allowing to transmit 65536 distinct editions of a message through the same stream before having to reset it.
- Expected number of replicas: This field contains the number of replicas *k*, $k^{\prime }$ on $k_{m}^{\prime \prime }$ that must be transmitted and, thus, expected to be received in a fault-free scenario. This field is 1 byte long, allowing for a maximum of 255 replicas per frame.

With this design, all the replicas of a given frame convey exactly the same information. Let us assume that we have a stream *S* with a stream identifier st through which we want to transmit *n* frames, $f_{st,1}$, $f_{st,2}$⋯$f_{st,n}$. Let us also assume that the frames transmitted through stream st must be replicated *m* times. The PTRF-related information conveyed by each replica $r_{st,id,j}$ of a frame $f_{st,id}$ will be the PTRF Ethertype 0x8815, the PTRF frame identifier id and the expected number of replicas *m*. Furthermore, all TSN frames convey the stream identifier st. Note that in approach C the number of replicas transmitted by each bridge via each link may vary. Therefore, the field called expected amount of replicas must be updated in each egress port of each bridge.

This structure of PTRF frames is fully compatible with standard TSN or Ethernet bridges. Whenever a standard bridge receives a PTRF replica, it checks the PTRF Ethertype and it interprets the field as a higher layer protocol. Therefore, the bridge treats the frame as it would treat any other regular frame, which allows us to use COTS bridges in approach A.

#### 6.4.3. Components

To enable the use of PTRF, the devices involved in the replication of frames and the elimination of replicas must include a series of new components. We must keep in mind during the next discussion that in approach A only end-systems implement PTRF, while bridges are standard COTS Ethernet bridges, and thus do not undergo any changes. On the other hand, in approaches B and C all end-systems and bridges implement PTRF. We next describe the new required components and we detail their deployment according to each approach.

**Figure 11:**
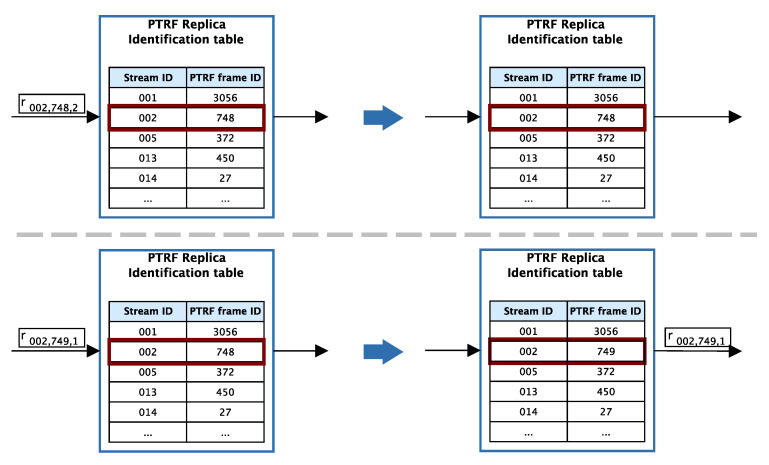
This figure shows the operation of the PTRF Replica Identification table. Specifically, on the top of the Figure, we see how the table processes a replica of a message edition that has previously been received. On the bottom, we see how the table processes a replica of a message edition that is received for the first time.

- PTRF replication selection table: As we have explained in Section 6.4.1, whenever a frame is transmitted, PTRF decides whether the frame must be replicated or not. This is done by consulting the PTRF replication selection table, which has two columns, (i) a list of all priorities, from 0 to 7 and (ii) the number of replicas to be transmitted for each priority. If the number of replicas for a given priority is 0, the PTRF mechanism is disabled for the streams of said priority. Therefore, frames of those streams are transmitted using the TSN standard transmission and frame format.
  In approach A there is a single table in each end-system. In approach B there is a single table in each end-system and bridge. In approach C there must be a different table in each eggress port of each end-system and bridge, to support different levels of replication for each link.
- Replica creation counter: This counter allows tracking the number of replicas of a given frame that have been created and queued. There must be one counter in each egress port of each PTRF-enabled device, in each approach. When the counter reaches the number of replicas *k*, $k^{\prime }$ or $k_{m}^{\prime \prime }$, it is reset and the same counter can be used to count the replicas of a new frame.
- PTRF replica identification table: This table stores the information of the last replica received through a stream by a PTRF-enabled device. This information is used to eliminate surplus replicas upon reception. Figure 11 shows the operation of this table. Whenever a PTRF-enabled device receives a replica, it must check whether this is the first received replica of a given message edition or not. To that, the device looks for the stream identifier up, to know to which stream the replica belongs to, stream 002 in the figure. Then, the device compares the PTRF frame identifier of the replica to the one stored in the table. If the stream identifier and the PTRF frame identifier in the replica are the same as the ones stored in the table, it means that this is not the first replica of that message edition received and, therefore, it can be discarded, as we see in the top of Figure 11. Otherwise, if the PTRF frame identifier of the table is different from the one in the replica, then this is the first replica that is received from the message edition. Thus, the table is updated with the new PTRF frame identifier and the replica is forwarded or delivered to the application, as we see in the bottom of Figure 11. We must note that we take advantage of the fact that bridges must store the stream identifier of all the streams that they forward to simply add the two-byte PTRF frame identifier.
- Replica reception counter: This counter is not used to support the operation of PTRF. Instead, this counter is devised to support the dynamic adaptation of the mechanism in the future. There must be one counter in each input port of each PTRF-enabled device, regardless of the approach. Specifically, the counter is increased each time a replica of a given frame is received. Thus, when compared to the expected amount of replicas *k*, $k^{\prime }$ or $k_{m}^{\prime \prime }$ this counter provides information about losses. The counter is reset whenever a new replica of the same or of a different frame is received. This information can be gathered by a management entity, such as the IEEE 802.1 Std Qcc CNC [23], to create new network configurations that properly adapt to the needs of the system.

## 7. PTRF Implementation

In this section we describe the implementation of a PTRF-enabled device which can be used as an end-system and as a bridge indistinctly. Furthermore, we describe the implementation of a fault injection device which has been designed and developed to support the validation and evaluation of the PTRF mechanism.

### 7.1. The PTRF-Enabled Device

The PTRF mechanism has been implemented on an already existing TSN bridge developed by System on Chip (SoC-e) [26]. Specifically, the mechanism is currently implemented as a firmware that operates on the MTSN Kit [27] developed by the company. The MTSN Kits used for this work had a version of the firmware (SoC-e continously upgrades this firmware) which implemented a series of TSN standards fully or partially, namely IEEE Std 802.1AS: Timing and Synchronization for Time-Sensitive Applications [20], IEEE Std 802.1Qav-2009: Forwarding and Queueing Enhancements for Time-Sensitive Streams [21], IEEE Std 802.1Qbv-2015: Enhancements for Scheduled Traffic [22] and IEEE Std 802.1Qcc-2018: Stream Reservation Protocol (SRP) Enhancements and Performance Improvements [23].

The PTRF mechanism is implemented on a Zynq UltraScale+ MPSoC [28]. This MPSoC can be roughly divided between a processing system and a programmable logic. In our experiments, the processing system executes the end-system application and it is in charge of producing the messages to be transmitted; while the programmable logic implements the MTSN switch IP with PTRF support. This allows us to use the MPSoC both as an end-system and as a bridge, both compatible with PTRF. Moreover, the implementation allows us to disable PTRF and use the MPSoCs as standard TSN devices.

**Figure 12:**
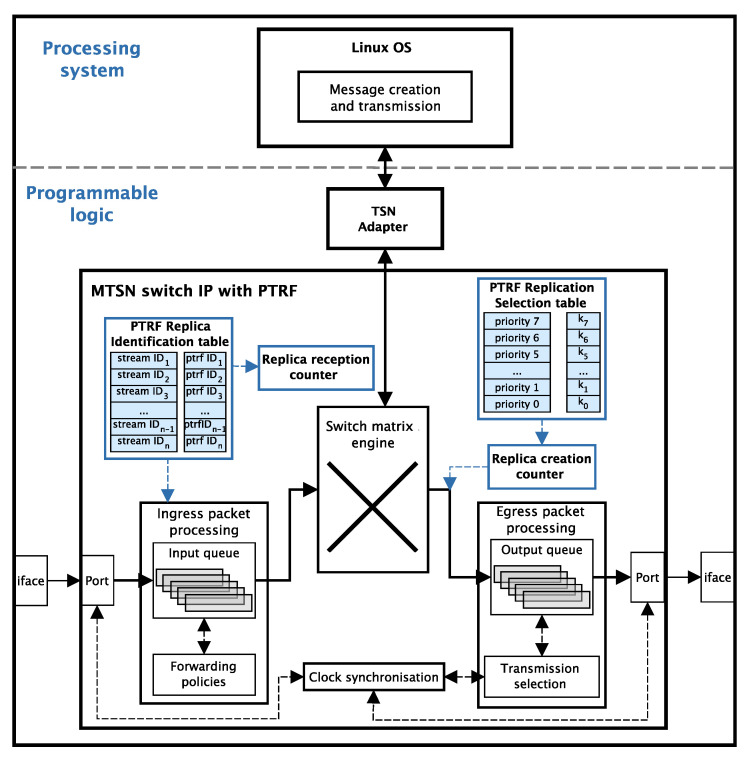
Basic architecture of the MPSoC that implements the PTRF mechanism. The PTRF components are highlighted in blue. Solid lines represent the path which frames follow whereas dashed lines represent management or configuration interactions.

Figure 12 depicts the architecture of the MPSoC with the Linux OS implemented in the processing system and the MTSN switch IP implemented in the programmable logic. The PTRF components described in Section 6.4.3 are highlighted in blue. Solid lines represent the path followed by the frames, whereas dashed lines represent management or configuration interactions. As we have mentioned, the MPSoC can be configured to operate as an end-system or a bridge.

When the MPSoC acts as an end-system, the application is executed in the Linux OS and the communication between the processing system and the programmable logic is activated. Messages created by the application are sent to the TSN adapter, which is implemented in the programmable logic and acts as a TSN network interface. Afterwards, the frame is forwarded to the adequate egress port using the switching logic and it is processed and transmitted as we describe next. On the other hand, when a frame is received through any of the ingress ports of the switch, the frame is passed to the application.

When the MPSoC acts as a bridge, the processing system is disabled and only the MTSN switch IP is active. Whenever a frame is received through a port, the PTRF replica identification table is consulted in order to detect replicas and decide whether it must be discarded or forwarded. If the replica must be forwarded, the bridge consults the forwarding policies and the replica is forwarded to the designated egress port.

Once a replica is forwarded to the egress port and before it is stored in the output queue, the PTRF replication selection table is consulted to decide whether the frame must be replicated or not. If the frame must be replicated, all replicas are created and stored in the corresponding output queue in a consecutive manner. No frames can be processed while the replicas are created, thus guaranteeing that all replicas are stored and transmitted subsequently. Once the transmission selection algorithm selects the corresponding queue, the replicas are transmitted through the egress port.

Finally, if PTRF is deactivated in the MPSoC, the programmable logic simply ignores the PTRF components and logic. Therefore, the MPSoC acts as a standard end-system or bridge, depending on the configuration. This is useful when implementing approach A, as only end-systems must be PTRF-devices, while bridges must be standard TSN.

It is important to note that we validated the implementation of the PTRF mechanism. That is, we carried out specific experiments to ensure that the PTRF mechanism is properly implemented. For the sake of succinctness the details of this validation are not included in this article.

### 7.2. The Fault Injection Device

As we have already explained, the PTRF mechanism is designed to tolerate temporary faults in the links. Therefore, to properly validate and evaluate PTRF we must study its behaviour in the presence of faults. We rely on prototype-based fault injection at the software level to carry out our evaluation [29]. This technique consists in using fault injection in a real prototype of the system to study its behaviour in the presence of faults. To do that, we use a device specifically designed to corrupt the desired frames (whether they are replicated or not) while they are being transmitted through the links in such a way that the receiver drops them thanks to the frames’ CRC.

This device has been developed by SoC-e on a Rely-RB time-aware redbox switch and it allows inspecting frames on-the-fly to (i) corrupt frames that match a predefined pattern, (ii) timestamp frames during their transmission through a link and (iii) measure the bandwidth consumption of a link in real time. This device has been developed ad hoc by the company and, thus, there is no public documentation or specification.

The Rely-RB is connected to the network as depicted in Figure 4a (LabTool). Basically, we place the Rely-RB between two devices (end-systems or bridges) that are exchanging information. Specifically, we connect one of the devices to port 0 of the Rely-RB and the other one to port 1. The Rely-RB carries out the designated operations (corrupt frames, timestamp or metering) on the frames that arrive through port 0 and forwards them through port 1. All these operations are done on-the-fly with nanosecond resolution and the overhead introduced by the device is negligible. In this way, the Rely-RB is transparent to the devices that communicate in the network.

Furthermore, the Rely-RB allows inspecting two different links simultaneously. Frames that arrive through port 2 are processed and forwarded through port 3. This is useful in our experiments as all the interfaces share a common clock. Therefore, we can use the Rely-RB as a common time reference to measure the end-to-end delay in our experiments.

We next describe the setups used to evaluate the PTRF mechanism, the experiments done and the results obtained.

## 8. Experimental Setup Characterisation

In this section we describe the hardware and software setup we use to carry out the experiments described in Section 9. In these experiments, we use up to four MTSN switches, two fault injection devices and a PC. One MTSN switch acts as an end-system that generates frames, while the rest of the MTSN switches act as pure TSN or PTRF-enabled TSN bridges. The fault injection devices are used to record the frames’ transmission or reception instants and to corrupt particular frames. We use the recorded timestamps to measure the end-to-end delay and jitter as described in Section 9. Finally, the PC acts as an interface to control all the other devices and it also captures the received frames with Wireshark [30], the network analyser.

It is important to note that in all of our experiments we use a line topology with a single path. This is so because we study the impact of time redundancy in the absence of spatial redundancy. Moreover, Ethernet eliminates loops in the network, creating a logical line topology even in mesh networks. On top of that, we only use a single transmitter and a single receiver, each of them placed in a different end of the line topology to maximise the number of hops between transmission and reception. Finally, even though TSN provides timing guarantees for up to six hops, we limit our experiments to four hops due to hardware restrictions.

Regarding the traffic, we transmit a single stream because we want to study the impact of PTRF and using interfering traffic could mask the impact of the mechanism and complicate the analysis unnecessarily. On the other hand, we carry out our experiments using TT traffic with priority 5. We do this because, many of the novel applications supported by TSN, such as Industry 4.0, autonomous driving or energy management strongly rely on TT traffic. Furthermore, TT traffic is usually subject to the highest timing and reliability requirements. Thus, even though PTRF can be used to replicate other types of traffic, we have decided to evaluate the mechanism using the most demanding type.

Finally, we use a 100Mbps network with different TAS configurations, to support different schedules for the TT traffic. We must note that we only use TAS as it is the only mechanism needed to transmit TT traffic. Furthermore, we use three different frame lengths (64, 782 and 1500 bytes) to be able to observe the impact of frame processing in the bridges. We use two, three and four replicas to study how the transmission of replicas impacts on the communications. We only use up to four replicas as previous works have shown this to be a sufficiently high number of replicas in other Ethernet-based reliable networks [31]. We run each experiment 1000 times, as we consider this to be a sufficiently large sample. We next describe the different experiments in detail and we discuss their results.

## 9. PTRF Evaluation and Results

As any other fault tolerance mechanism, PTRF has an impact on the network performance. This is because the transmission of replicas implies an increase in the resources required to exchange a single message, e.g., time or bandwidth. As we have already mentioned, we have evaluated PTRF from three different points of view, namely (i) impact on the end-to-end delay, (ii) introduction of jitter in the transmission and (iii) impact on the bandwidth consumption. We carry out all of our measurements in the layer 2 of the network architecture for two reasons. First, all the mechanisms proposed by the TSN TG operate at the layer 2 and PTRF is also designed to operate in such layer. Second, we carry out a sensitivity analysis to study the behaviour of PTRF when operating in networks with different characteristics. Therefore, our study must be application independent and, thus, we abstract the operation of the application.

It is important to note that in this article we do not measure the impact of using time redundancy in a specific application. Instead, we study the overhead introduced by the devices that implement PTRF and we carry out a sensitivity analysis. This is so because the actual impact on the temporal behaviour and the bandwidth depends on the redundancy level and the network utilisation of each application. At any rate, PTRF is only to be used in systems that require a certain level of reliability, even if that means reducing the response time or the bandwidth available for noncritical traffic.

We have carried out each of the following experiments for three different types of networks: a standard TSN network, a network implementing approach A and a network implementing approach B. As the implementation of approaches B and C is the same, we do not carry out any specific experiments to measure the difference between these approaches. Furthermore, a study on the gains of using approach C over approach B in dynamic networks is out of the scope of this paper and is left as future work.

We next describe the experiments done and the results obtained.

### 9.1. End-to-End Delay

We carry out two different sets of experiments to study the impact of PTRF on the end-to-end delay. The target of the first set of experiments is to measure the overhead that using PTRF causes on the end-to-end delay of a single frame in the absence of faults. To that, we transmit a single frame in each one of the networks with no replication, we measure the end-to-end delay and we compare the results obtained with approaches A and B to those obtained with standard TSN to measure the overhead.

We must note that we define the end-to-end delay of a set of replicas as the time elapsed since the first replica starts being transmitted until the first correct replica reaches the receiver. Since in this set of experiments we transmit a single frame in the absence of faults, the end-to-end delay is the time elapsed since the frame is transmitted until it is received.

**Figure 13:**
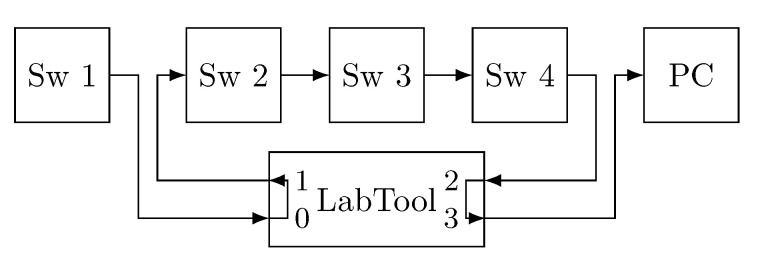
Topology used to measure the end-to-end delay in the absence of faults.

Figure 13 shows the topology. The first MTSN switch (Sw 1) acts as an end-system and transmits the frames, which are forwarded on the other three MTSN switches until they are captured with Wireshark on the right-hand PC. The fault injection device (LabTool) is used to record the frames’ timestamps. The difference between the time recorded on port 3, when the frame reaches the PC, and port 0, when the frame leaves the end-system, is the end-to-end delay of the frames. TAS is configured in each device to allow all priority 5 TT traffic pass and block the rest of the traffic, preventing interference. Furthermore, frames are transmitted with an interframe gap of 2 ms to avoid queuing delays.

As we have mentioned, we study standard TSN, approach A and approach B and we use three different frame lengths (64, 782 and 1500 Bytes), producing a total of nine experiments, in each of which 1000 consecutive nonreplicated frames are exchanged. Table 2 shows the mean, the standard deviation, the maximum and the minimum end-to-end delay. As expected, we can see that the frame length highly impacts the end-to-end delay, as the time required to transmit and process a large frame is significantly higher than for a short one. On the contrary, we observe that the impact of the PTRF mechanism in the end-to-end delay is actually low.

Specifically, we see that the difference in the end-to-end delay between PTRF (both following approach A or B) and TSN is lower than 125 ns, even for the minimum end-to-end delay, i.e., the overhead caused by PTRF is lower than 125 ns. Moreover, we see that PTRF does not cause significant variations in the end-to-end delay in the absence of faults, as we can see observing the standard deviation. Thus, we can conclude that the overhead introduced when the PTRF mechanism is implemented does not pose a threat to the timeliness of the system.

Now that we have measured the impact that the PTRF mechanism has on the end-to-end delay, we want to measure the impact of sending replicas. As we have anticipated at the beginning of this section, we have carried out a second set of experiments. Specifically, in this set we measure the maximum end-to-end delay when we transmit several replicas in the presence of faults.

**Figure 14:**
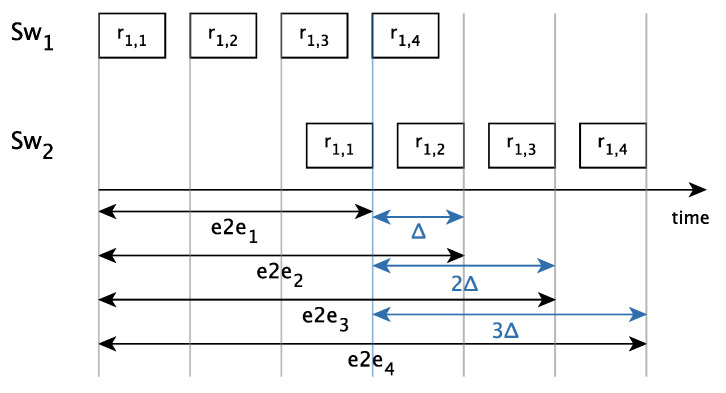
This figure shows the end-to-end delay of four replicas transmitted through a link between two bridges (Sw1 and Sw2). Specifically, the figure shows the transmission of four replicas with their respective maximum end-to-end delays marked with black arrows, i.e., e2e1 is the maximum end-to-end delay when transmitting a single replica, e2e2 is the maximum end-to-end delay when transmitting two replicas, etc. Moreover, the blue arrows show the variation between the end-to-end delays, a.k.a. jitter.

Figure 14 shows the end-to-end delay of four replicas transmitted through a single link, more specifically the link between Sw1 and Sw2 in Figure 13. As we have explained, when we talk about a set of replicas, we measure the end-to-end delay as the time elapsed since the first replica starts being transmitted until the first correct replica is completely received. Therefore, the maximum end-to-end delay corresponds to only receiving the last replica ($r_{1,4}$ in Figure 14), i.e., losing all replicas but the last one due to faults.

**Figure 15:**
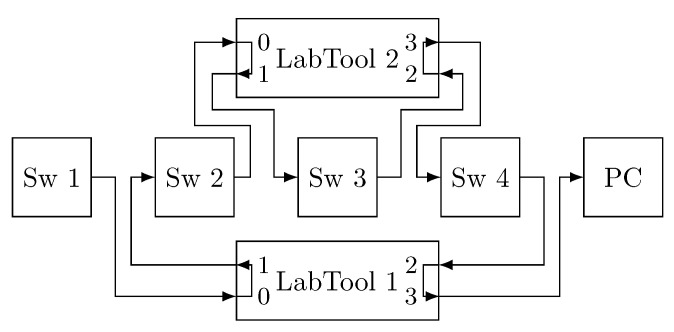
Topology used to measure the end-to-end delay and the jitter in the presence of faults.

We use the network configuration shown in Figure 15 to measure the maximum end-to-end delay. As in the previous experiment, the first MTSN switch is used as an end-system to transmit frames and the other three switches are used as TSN or PTRF bridges. LabTool 1 is used to timestamp frames and to inject errors on predefined frames, while LabTool 2 is used only as error injection device. As we can see, LabTool 1 is connected to Sw1 and to the PC to have a common time reference when measuring the end-to-end delay.

In this case, we only study approach A and approach B, as TSN does not provide time redundancy. Thus, comparing the approaches to TSN would result in an unfair comparison, as TSN would always be faster but, in contrast, cannot provide the same level of fault tolerance.

As in the previous experiment, we use three different frame lengths (64, 782 and 1500 Bytes) and we transmit 1000 different frames. The end-system (Sw 1) transmits two, three and four replicas of each frame, resulting in nine experiments for each network. In approach A only the end-system creates replicas and LabTool 1 is configured to introduce errors in all replicas but the last one, only in the first link. The bridges Sw 2, Sw 3 and Sw 4 simply forward the correct frames or replicas and, thus, LabTool 2 is configured to pass frames without errors.

On the other hand, in approach B all the switches use PTRF and all of them are configured to replicate two, three and four times depending on the experiment. In this case, Labtool 1 and LabTool 2 are configured to introduce errors in all replicas but the last one in all links. Each bridge receives the only correct replica and produces a new set of replicas. We introduce errors in all replicas but the last one in all links to achieve the maximum end-to-end delay in the multihop network.

Table 3 shows the mean, the standard deviation and the maximum values for the maximum end-to-end delay when transmitting two, three and four replicas using approaches A and B. As expected, the maximum end-to-end delay increases with the number of replicas transmitted and it is higher in approach B than in approach A. Nevertheless, it is important to note that the number of fault scenarios that can be tolerated also increases with the number of replicas and it is also higher using approach B than using approach A, as we showed in [13]. Moreover, we can observe that the standard deviation is really low when the frame length is short, but it significantly increases for medium and large frames, which means that the deviation in the end-to-end delay is higher when using time redundancy with larger frames.

**Figure 16:**
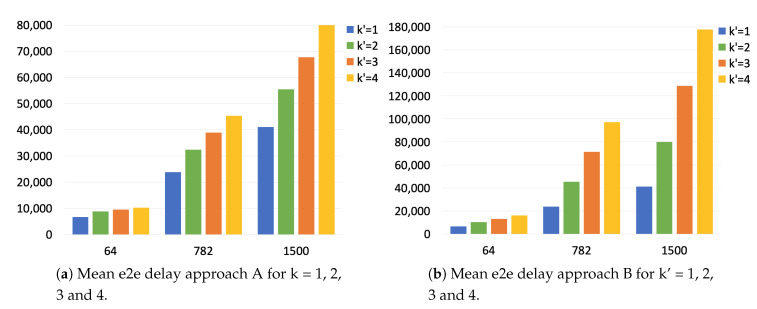
Mean value for the maximum end-to-end delay for approaches A and B with *k* = *k*’ = 1, 2, 3 and 4. The X axis represent the frame length in bytes, while the Y axis represent the end-to-end delay in nanoseconds.

We now compare these results to the ones obtained in the previous experiment ( Table 2). Figure 16 shows the mean value for the maximum end-to-end delay for approaches A and B when transmitting one, two, three and four replicas. Observing the results of approach A we see that the difference in the end-to-end delay of k=1 with k=2 is around 2 μs higher than the difference of k=2 with k=3 and k=3 with k=4, regardless of the frame length. We can thus conclude that there are two different contributions to the overhead introduced when transmitting replicas: (i) one contribution is common to all the cases and could be caused by the regular forwarding and error detection mechanisms, (ii) another contribution that varies depending on the number of replicas transmitted and their length. In any case, we can see that the end-to-end delay is always under 80 μs.

Regarding approach B, we see that maximum the end-to-end delay increases considerably with the number of replicas transmitted regardless of the frame length. This is because in approach B the whole set of replicas is transmitted in every link, even though some replicas are corrupted during transmission. Furthermore, since in this experiment we corrupt all replicas but the last one, each bridge must wait for the last replica in order to generate the new set. Since this is repeated in each hop, the impact is considerably higher than in approach A. In any case, we see that the end-to-end delay is under 80 μs for frames of small and medium length and under 180 μs for large frames.

### 9.2. Transmission Jitter

Timeliness is crucial to guarantee the correct operation of hard real-time systems. In fact, a factor that can negatively impact the correct behaviour of a system is the jitter in the communication. Jitter can roughly be defined as the variation on the end-to-end delay between the transmission of several frames through a path or stream. This variations can be caused by differences in the queuing and processing times. In PTRF, we have to also consider the variations in the queuing and processing times that replication introduces, as well as the time required for the creation of replicas.

**Figure 17:**
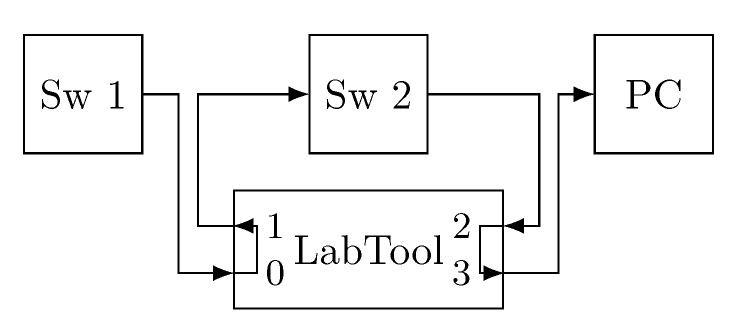
Topology used to measure the jitter in the absence of faults.

We carry out a first set of experiments to measure the jitter. In the first set of experiments we use the simple topology depicted in Figure 17 with a single end-system (Sw 1) and a single bridge (Sw 2). In this way we can isolate the impact of PTRF from other queuing and processing variations. The end-system transmits 2, 3 and 4 replicas. We then compare the time required to transmit the last replica to the time required to transmit the first one to obtain the jitter. We do this for frame lengths of 64, 782 and 1500 bytes. We carry out this experiment in the absence of faults as we want to compare the jitter introduced by the creation and processing of replicas.

Table 4 shows the results of this first experiment. The first thing we notice observing the results is that the jitter measured is 0 when *k* = $k^{\prime }$ = 2 and the frame length is 64 B, probably because the variation in the delay could not be measured by our equipment. Since we only have one hop and the cable and frame lengths are short, the jitter is clearly negligible in this case for both approaches.

Observing the rest of our results, we clearly see that the jitter increases with the frame length and the number of replicas. Moreover, we can see that the mean jitter measured for approach B is slightly higher than for approach A in most cases, probably due to the fact that the bridge (Sw 2) must eliminate and create the new set of replicas. This is also reflected in the standard deviation, which is mostly higher for approach B than approach A, which means that the jitter itself suffers a higher variation in the former.

Nevertheless, in PTRF we have to consider an additional level of jitter, as the end-to-end delay of a frame varies depending on which correct replica reaches its destination first in the presence of faults. More specifically, we measure this level of jitter as the difference between the minimum and the maximum end-to-end delay, i.e., the difference in the end-to-end delay when the first replica is correctly received and when only the last replica is correctly received. We carry out these experiments in the presence of faults to force the loss of all replicas but the last one.

Figure 14 depicts the jitter introduced by PTRF when transmitting a different number of replicas in the presence of faults. Let us assume that Sw1 only transmits two replicas, $r_{1,1}$ and $r_{1,2}$. Let us also assume that in one transmission $r_{1,1}$ reaches Sw2 with end-to-end delay $e2e_{1}$. Let us now assume that in another transmission, $r_{1,1}$ does not reach Sw2, but $r_{1,2}$ does. In this case, the end-to-end delay is $e2e_{2}$. The difference between both end-to-end delays is the jitter, indicated with Δ in Figure 14. We also see in Figure 14 the jitter when transmitting three replicas, 2Δ, and four replicas, 3Δ.

Thus, we carry out a second set of experiments. We now measure the jitter in the presence of faults using the network topology depicted in Figure 15. In this way, we can study the impact of using a larger network and we can measure this new level of jitter introduced by PTRF.

Table 5 shows the measured jitter for each approach, with the different frame lengths and number of replicas. If we take a close look to the results obtained for $k=k^{\prime }=2$ replicas and frame length of 64 B, we see that the maximum jitter is 2.2 μs for approach A and 3.7 μs for approach B. Therefore, we can corroborate that the jitter for a single hop was too low to be measured by our equipment.

Observing the overall results, we can see that, just like the end-to-end delay, the jitter is greatly affected by the frame length and number of replicas. Moreover, we also see that the jitter introduced by approach B is significantly higher than by approach A. This is because in approach B there is a contribution to the jitter in each link, while in approach A there is only the contribution in the first link.

In any case, we can see that the maximum measured jitter for a 4-hop network and four replicas using approach A is under 40 s, even for the largest frames. On the other hand, we see that the maximum jitter for approach B is considerably higher, around 137 μs. Again, it is important to note that the number of fault scenarios tolerated by approach B using the same number of replicas is also considerably higher than by approach A. Nevertheless, these results show that when the frame length is large and the number of replicas must be high, approach A is a more suitable solution than approach B for jitter-sensitive applications.

### 9.3. Bandwidth Overhead

Bandwidth consumption is one of the main concerns in the development of novel converged networks for the applications targeted by TSN. For this reason, it is important to minimise the impact that fault tolerance mechanisms have on the bandwidth consumption. Moreover, it is of utmost importance to quantify this impact. We carry out two different experiments to measure the impact that PTRF has on bandwidth.

To measure how PTRF impacts bandwidth, we measure the overhead introduced by creating replicas. To that, we carried out a first experiment to compare the time required to transmit a set of frames to the time required to transmit a set of replicas. That is, we measured the time required to transmit 1000 frames to the time required to transmit 1000 replicas of a single frame. Nevertheless, during this experiment we observed that the time required to produce a replica is significantly shorter than the time required to produce a new frame. This is due to the fact that frames are produced by the application and processed by the software stack, while replicas are created directly in the network level. Therefore, this experiment did not allow us to quantify the bandwidth overhead.

Therefore, we carried out a second set of experiments in which, instead of sending replicas of a single frame, we replicated different frames using the network depicted in Figure 13. In approach A the end-system (Sw 1) transmits two, three and four replicas while the TSN bridges (Sw 2, 3, 4) only forward them. In Approach B, the end-system and the PTRF bridges produce the same number of replicas, which go from two to four. Moreover, we carry out each experiment with frame lengths of 64, 782 and 1500 Bytes to take into account the different processing times, resulting in 21 experiments.

In order to measure the impact on the bandwidth, we count how many frames (with their corresponding replicas) can be transmitted within a time slot using TSN, approach A and approach B. The lower the number of frames within a slot, the higher the impact on bandwidth of the mechanism. We believe that measuring the impact on bandwidth in a per-slot basis is a good approach for TSN networks, because the communication is divided in time slots which means that the schedule is also calculated in a per-slot basis.

We use TAS, which we described in Section 3, to configure the time slots for all priorities. Specifically, we use three different configurations for priority 5 (i) 1 ms open and 5 ms closed, (ii) 10 ms open and 5 ms closed and (iii) 20 ms open and 5 ms closed; where open means that frames can be transmitted while closed means that frames cannot be transmitted. The time during which the gate is closed allows us to delimit the different slots and count the number of frames that the network is going to be able to effectively exchange within each slot. The gates of the rest of priorities are always configured to be closed so no frames from a different priority can be transmitted during the experiments.

We decided to use slot sizes of 1, 10 and 20 ms, as we consider that these sizes are diverse enough to study the behaviour of PTRF under different circumstances, while still being realistic, e.g., 1 ms for the transmission of control traffic and 20 ms for the transmission of multimedia traffic.

Table 6 shows the mean number of frames that the network can effectively exchange within a time slot for TSN, approach A and approach B. Note that since we have calculated the mean, we show the results with three decimals, even though the number of frames exchanged within a slot is, obviously, an integer number. As expected, the number of frames that can be exchanged within a slot increases with the size of the slot and decreases with the frame length. Regarding the impact of PTRF, we can see that the overhead when the time slot is small is barely noticeable, regardless of the frame size. With a medium-size slot TSN can convey 1 more frame per slot than approaches A and B and with large slots TSN can convey up to two frames more per slot. Thus, we can see that the impact on bandwidth of using PTRF is low. Furthermore, we can see that the difference between approaches A and B is negligible for all slot sizes and frame lengths studied.

### 9.4. Final Discussion

The design of a fault-tolerant system or mechanism is always a trade-off between reliability and performance. Nevertheless, in certain scenarios the use of such mechanisms is essential, and even mandatory, to guarantee the correct operation of the system. In these cases, the target is to design fault tolerance mechanisms that have the lowest impact on the systems’ performance. Observing the results obtained, we can conclude that the PTRF mechanism is a suitable solution to provide time redundancy to TSN networks.

On the one hand, we have seen that the impact of PTRF on the end-to-end delay is negligible in the absence of faults, always under 125 ns. Furthermore, we have also studied the maximum end-to-end delay in the presence of faults, which corresponds to losing all replicas except for the last one during transmission. The maximum end-to-end delay was always under 80 s for approach A and under 180 s for approach B, even when transmitting four replicas of 1500 B. Thus, we think that both approaches provide acceptable values for the maximum end-to-end delay, with approach A providing the lowest upper bound of the two.

Regarding jitter, we have observed that the jitter introduced by the creation and processing of replicas in the absence of faults is under 40 s even for the largest frames and highest number of replicas. Furthermore, the difference between approaches A and B is negligible. On the other hand, we have studied the jitter introduced by the transmission of replicas in the presence of faults. In this scenario we observe that the jitter for approach A was always under 40 s, and the jitter for approach B was under 140 s, even when transmitting four replicas of 1500 B. Observing all the results, we can conclude that both approaches are adequate when the frame length is short or the number o replicas is low. Nevertheless, for larger frames or higher number of replicas, approach A is a more adequate solution than approach B, especially for time-sensitive applications.

We have also seen that the impact of PTRF in the bandwidth is low. In fact, we have seen that generating replicas requires considerably less time than generating frames, as replicas are created in the second layer of the communication stack, while frames are generated in the application layer. On the other hand, we have seen that the overhead introduced by the process of creating replicas is negligible for short frames and small time slots. Furthermore, the difference on the number of frames that can be exchanged in a slot is also low when the frame length or the slot size increase, i.e., up to two more frames exchanged per slot in TSN than in PTRF.

For all these reasons, we think that PTRF is an adequate solution to tolerate temporary faults in the links of TSN-based networks, even for time-sensitive applications with tight timing requirements. We think that the impact that approaches A and B have on the performance of the system is acceptable for applications that require a high level of reliability.

## 10. Conclusions

The TSN TG has developed a series of standards to provide Ethernet with enhanced services, such as support for hard real-time traffic, online management of the traffic and increased reliability by means of fault tolerance. With these advancements, the TG aims at creating the mechanisms to turn Ethernet into the standard network technology of the future. In fact, the TSN standards could be used to build the network infrastructure of novel applications such as Industry 4.0, autonomous driving or smart energy management and distribution. Many of these novel applications have stringent timing and reliability requirements, imposed by the environment in which they operate. To increase the reliability, the TSN TG standardised a spatial redundancy mechanism to tolerate faults in Ethernet networks. Nevertheless, TSN does not propose any time redundancy mechanisms in the layer 2 of the network architecture for tolerating temporary faults in the links.

Since temporary faults in the links are the most likely to happen in any network, and time redundancy is specially suitable for tolerating this type of faults, we have proposed the so-called PTRF mechanism. PTRF is a time redundancy mechanism which consists in transmitting several copies of each frame in a preventive manner to maximise the chances of at least one copy reaching its destination even in the presence of temporary faults. More concretely, we have proposed three different approaches to implement PTRF. Specifically, we have proposed (i) approach A: where only end-systems replicate frames, (ii) approach B: where both end-systems and bridges replicate frames with the same level of replication and (iii) approach C: where both end-systems and bridges replicate frames, but the level of replication can vary depending on the forwarding link.

In this work we present for the first time a detailed description of the design of PTRF. In fact, this is the first time that we describe the types of faults that we want to tolerate and how we assume the network behaves in front of them; the design rationale that we followed to propose the three approaches of PTRF; the operation of the three approaches of the mechanism, their complete infrastructure and their qualitative comparison. Furthermore, we present the implementation of the mechanism in a real prototype of a TSN-based network developed by the industry and we carry out an experimental evaluation of the two first approaches from the point of view of the end-to-end delay, the jitter and the bandwidth.

Specifically, we have measured the end-to-end delay and jitter both in the absence and in the presence of temporary faults and we have evaluated the bandwidth overhead in the absence of faults. We must note that this work does not aim at evaluating PTRF in a specific network or application, but the aim is evaluating the PTRF mechanism itself and provide a sensitivity analysis. To that, we have carried out the experiments with different frame lengths and number of replicas to evaluate their impact on the performance of the two approaches (A and B) under experimentation.

Regarding the end-to-end delay, we have seen that the overhead introduced by PTRF in the absence of faults is negligible, always under 125 ns, for both approaches. In the presence of faults, the maximum end-to-end delay with approach B is significantly higher than with approach A, 180 s for approach B and 80 s for approach A. In any case, we think that both approaches provide acceptable values for the maximum end-to-end delay.

Regarding the evaluation of the jitter, we have seen that the jitter introduced by the creation and processing of replicas in the absence of faults was always under 40 s, even for the largest frame sizes and number of replicas. Nevertheless, in the presence of faults the jitter increases considerably with the frame length and the number of replicas. Furthermore, the jitter is considerably worse for approach B than A, around 100 s worse. Thus, approach A may be a more suitable solution than approach B for time-sensitive applications that are vulnerable to jitter.

Regarding the bandwidth, we have observed that the impact that creating replicas has on the bandwidth consumption is almost negligible, regardless of the approach, the frame length and the number of replicas. This is probably due to the fact that creating replicas is considerably faster than creating new frames. This is because frames are created in the application layer and must traverse the whole system stack before being transmitted, while replicas are created in the network layer, which is implemented in hardware.

Finally, designing a fault tolerance mechanism is a trade-off between reliability and performance. Since certain applications require the use of fault tolerance mechanisms, our objective was to develop a fault tolerant mechanism with a low impact on the network performance. Analysing the results that we have obtained, we can conclude that PTRF is an adequate solution to tolerate temporary faults in the links of TSN-based networks, with different approaches to adapt to the needs of different network architectures and applications.

## Figures and Tables

**Table 1:** Number of frames received through each port in the absence of faults, presence of temporary faults only and presence of temporary and permanent faults.

Experiment	Number of Received Frames			Number of Lost Frames
	Interface 1	Interface 2	Interface 3	
Exp I	1000	1000	—	0
Exp II	990	1000	—	0
Exp III	990	0	—	10
Exp IV	990	0	1000	0

**Table 2:** End-to-end delay when transmitting a single frame using TSN, approach A and approach B through a 4-hop network.

Network	Frame	End-to-End Delay (ns)			
	Length (B)	Mean	std	Max	Min
TSN	64	6487.600	15.704	6534	6435
	782	23,743.979	15.704	23,787	23,697
	1500	40,975.512	15.503	41,022	40,923
App A	64	6631.830	15.989	6687	6588
	782	23,863.720	15.222	23,904	23,814
	1500	41,095.400	16.498	41,157	41,049
App B	64	6630.509	16.538	6696	6570
	782	23,863.204	16.520	23,913	23,823
	1500	41,095.432	15.581	41,139	41,049

**Table 3:** Maximum end-to-end delay in nanoseconds when transmitting two, three and four replicas using approach A and approach B.

Network	Frame	*k* = $k^{\prime }$ = 2			*k* = $k^{\prime }$ = 3			*k* = $k^{\prime }$ = 4		
	Length (B)	Mean	std	Max	Mean	std	Max	Mean	std	Max
App A	64	8778.74	18.99	8829	9513.73	18.48	9576	10,250.45	19.31	10,314
	782	32,412.10	705.81	32,544	38,912.56	612.81	39,033	45,392.19	611.56	45,522
	1500	55,454.69	386.92	55,521	67,679.37	387.06	67,752	79,916.40	19.86	79,965
App B	64	10,250.16	18.03	10,314	13,195.39	18.63	13,248	16,138.32	17.58	16,191
	782	45,409.83	507.82	45,495	71,350.47	354.20	71,424	97,250.73	521.15	97,353
	1500	79,890.84	547.79	79,974	12,8799.66	387.34	128,871	17,7681.99	583.65	177,768

**Table 4:** Mean, standard deviation and maximum jitter when transmitting two, three and four replicas using approach A and approach B in a one-hop network. All the results are in nanoseconds.

Network	Frame	*k* = $k^{\prime }$ = 2			*k* = $k^{\prime }$ = 3			*k* = $k^{\prime }$ = 4		
	Length (B)	Mean	std	Max	Mean	std	Max	Mean	std	Max
App A	64	0	0	0	736.030	4.058	756	1472.332	4.834	1494
	782	6479.855	3.522	6498	12,894.142	682.271	15,129	19,354.956	1156.110	25,920
	1500	12,224.751	5.803	12,249	24,412.945	701.358	26,631	36,555.708	1410.492	36,702
App B	64	0	0	0	736.164	3.824	756	1472.058	4.627	1485
	782	6460.380	354.751	6489	12,920.900	500.816	12,969	19,404.496	475.906	19,449
	1500	12,211.308	386.568	12,240	24,447.141	7.804	24,462	36,633.248	687.231	36,702

**Table 5:** Mean, standard deviation and maximum jitter when transmitting two, three and four replicas using approach A and approach B in a four-hop network In the presence of faults. All the results are in nanoseconds.

Network	Frame	*k* = $k^{\prime }$ = 2			*k* = $k^{\prime }$ = 3			*k* = $k^{\prime }$ = 4		
	Length (B)	Mean	std	Max	Mean	std	Max	Mean	std	Max
App A	64	2146.914	24.570	2232	2882.125	25.096	2961	3618.641	25.084	3708
	782	8543.211	728.160	8694	15,051.944	596.498	15,174	21,524.725	630.428	21,681
	1500	14,358.351	398.471	14,445	26,583.137	399.794	26,667	38,821.610	389.461	38,889
App B	64	3618.333	23.894	3690	6563.565	24.271	6642	9506.493	23.673	9576
	782	21,546.346	507.302	21,663	47,485.637	366.145	47,574	73,387.060	521.007	73,512
	1500	38,794.171	563.057	38,898	87,703.380	398.456	87,804	136,586.633	583.833	136,692

**Table 6:** Mean number of number of frames that the network can effectively exchange within a slot using TSN, approach A and approach B, with different slot sizes and frame lengths.

Network	Frame	Number of Frames per Slot		
	Length (B)	1 ms|5 ms	10 ms|5 ms	20 ms|5 ms
TSN	64	23.827	163.669	322.587
	782	23.607	162.750	318.478
	1500	20.246	161.166	317.321
App A	64	23.534	162.287	321.682
	782	23.798	161.574	316.459
	1500	19.978	160.197	316.957
App B	64	23.937	162.820	321.550
	782	23.821	161.554	316.462
	1500	20.139	160.567	314.226

## Data Availability

Data can be accessed by requesting it to the corresponding author.
